# Chemogenetic activation of CRF neurons as a model of chronic stress produces sex-specific physiological and behavioral effects

**DOI:** 10.1038/s41386-023-01739-5

**Published:** 2023-10-13

**Authors:** Kristen R. Montgomery, Morgan S. Bridi, Lillian M. Folts, Ruth Marx-Rattner, Hannah C. Zierden, Andreas B. Wulff, Emmanuela A. Kodjo, Scott M. Thompson, Tracy L. Bale

**Affiliations:** 1 Department of Pharmacology, University of Maryland School of Medicine, Baltimore, MD 21201 USA; 2 Center for Epigenetic Research in Child Health and Brain Development, University of Maryland School of Medicine, Baltimore, MD 21201 USA; 3 Neuroscience Graduate Program, University of Colorado Anschutz Medical Campus, Aurora, CO 80045 USA; 4 Department of Physiology, University of Maryland School of Medicine, Baltimore, MD 21201 USA; 5 Department of Psychiatry, University of Colorado Anschutz Medical Campus, Aurora, CO 80045 USA

**Keywords:** Stress and resilience, Experimental organisms

## Abstract

Trauma and chronic stress exposure are the strongest predictors of lifetime neuropsychiatric disease presentation. These disorders often have significant sex biases, with females having higher incidences of affective disorders such as major depression, anxiety, and PTSD. Understanding the mechanisms by which stress exposure heightens disease vulnerability is essential for developing novel interventions. Current rodent stress models consist of a battery of sensory, homeostatic, and psychological stressors that are ultimately integrated by corticotropin-releasing factor (CRF) neurons to trigger corticosteroid release. These stress paradigms, however, often differ between research groups in the type, timing, and duration of stressors utilized. These inconsistencies, along with the variability of individual animals’ perception and response to each stressor, present challenges for reproducibility and translational relevance. Here, we hypothesized that a more direct approach using chemogenetic activation of CRF neurons would recapitulate the effects of traditional stress paradigms and provide a high-throughput method for examining stress-relevant phenotypes. Using a transgenic approach to express the Gq-coupled Designer Receptor Exclusively Activated by Designer Drugs (DREADD) receptor hM3Dq in CRF-neurons, we found that the DREADD ligand clozapine-N-oxide (CNO) produced an acute and robust activation of the hypothalamic-pituitary-adrenal (HPA) axis, as predicted. Interestingly, chronic treatment with this method of direct CRF activation uncovered a novel sex-specific dissociation of glucocorticoid levels with stress-related outcomes. Despite hM3Dq-expressing females producing greater corticosterone levels in response to CNO than males, hM3Dq-expressing males showed significant typical physiological stress sensitivity with reductions in body and thymus weights. hM3Dq-expressing females while resistant to the physiological effects of chronic CRF activation, showed significant increases in baseline and fear-conditioned freezing behaviors. These data establish a novel mouse model for interrogating stress-relevant phenotypes and highlight sex-specific stress circuitry distinct for physiological and limbic control that may underlie disease risk.

## Introduction

The ability to respond to environmental and homeostatic perturbations is critical for survival, and accordingly, the stress response is highly conserved across species [1–8]. In response to stress, corticotropin-releasing factor (CRF) neurons, including in the paraventricular nucleus of the hypothalamus (PVN), prefrontal cortex, hippocampus, basolateral and central amygdala (BLA, CeA), and bed nucleus of the stria terminalis (BNST), modulate physiological, behavioral, and endocrine responses [9–20]. Limbic and cortical afferents converge onto CRF neurons in the PVN to initiate the hypothalamic-pituitary-adrenal (HPA) stress response, resulting in glucocorticoid secretion from the adrenal gland into circulation [17, 21–26].

Chronic stress and activation of CRF neurons are strongly linked to neuropsychiatric disorder development [17, 19, 21, 27–38]. Rodent models are commonly used to investigate the mechanisms by which chronic stress exposure contributes to risk as they recapitulate many of the behavioral and physiological effects seen in humans. Most models involve a battery of psychological, sensory, and homeostatic stressors, and while effective in inducing stress responses, the type, duration, and timing of exposure varies widely across research labs and produces an array of behavioral and physiological changes that rely on the individual animal’s perception of and response to the stress, often resulting in variability between cohorts of animals even within a single lab [39–45]. Even widely used models, such as chronic variable or unpredictable stress, are often modified to fit the needs of individual groups. Sex differences in the efficacy of some models add an additional complication, hindering our collective ability to uncover important sex-specific mechanisms in stress-related disorders or rigorously assess novel treatment efficacy [46–50].

To minimize variability and create a high-throughput system for investigating stress-relevant disorders, we developed a mouse model using the Gq-coupled Designer Receptors Exclusively Activated by Designer Drugs (DREADD) hM3Dq to selectively activate all CRF neurons in a temporally defined window and used palatable cookie dough treats to administer CNO, eliminating the need for daily intraperitoneal (i.p.) injections [51]. The DREADD ligand clozapine-N-oxide (CNO) activates the canonical Gq pathway, triggering neuronal discharge [51, 52]. Viral injection is the most common method for DREADD expression; however, notable drawbacks include variability in injection site placement, labor cost, and latency of expression. To minimize this variance, we utilized a transgenic strategy in which CRF-Cre mice were crossed with mice expressing a floxed DREADD hM3Dq gene. Here, we show that chemogenetic CRF-neuron activation effectively initiates the HPA axis stress response and that repeated, chronic activation induces sex- and brain region-specific effects.

## Methods

Detailed methods are presented in Supplementary Materials

### Animals

Adult (10–20 weeks) male and female mice heterozygous for the CRF-Cre transgene and hM3Dq transgene (CRF-Cre^+^/^-^ X DREADD^+^/^-^, defined as DREADD + ) or heterozygous for the CRF-Cre transgene and wild-type for the hM3Dq transgene (CRF-Cre^+^/^-^ X DREADD^-^/^-^, defined as DREADD-) were used for DREADD studies. Adult (8–9 weeks) male and female C57BL/6 J mice were used for chronic multimodal stress. All animal experiments were approved by the University of Maryland Baltimore Institutional Animal Care and Use Committee and conducted in accordance with the National Institutes of Health Guide for the Care and Use of Laboratory Animals.

### CNO administration

For i.p. injections, clozapine N-oxide (CNO) dihydrochloride (Hello Bio, catalog #HB6149) was prepared in 0.9% saline at 1 mM. Cookie dough treats were prepared at 0.1 mg CNO/g dough (Transgenic Dough Diet, Bio-Serv catalog #S3472, sugar cookie (Pillsbury), or Reese’s Peanut Butter (Pillsbury)).

### Hypothalamic-Pituitary-Adrenal response to CNO and acute restraint stress

HPA axis reactivity to an i.p. CNO injection or 15 min restraint was measured as previously described [53]. 10 uL of blood was collected from a tail snip at specified time points, and plasma corticosterone levels were measured by ^125^I-Corticosterone radioimmunoassay (MP Biomedicals, catalog #07120103).

### Chronic multimodal stress

8–9 week male and female C57BL/6 J mice were subjected to stress for 14 days as described [54, 55]. Stress consisted of restraint in a 3D printed restraint tube (Ender 3 printer, Creality), in a cage tilted 30° while being subjected to white noise, strobe lights, and predator odor (fox urine, Trap Shack Company). These mice were only examined for changes in the von Frey filament test.

### Immunohistochemistry and thymus collection

3 h after a single CNO injection, mice were anesthetized and transcardially perfused and the thymus was dissected out. c-Fos immunohistochemistry was performed using c-Fos (1:2500, Synaptic Systems #226–308) and anti-guinea pig (1:200, Alexa Fluor 568, Thermo Fisher, #11075) antibodies and Hoechst counterstain (1:2000, Thermo Fisher, #33342). c-Fos density was normalized to Hoechst for each section. For HA immunohistochemistry, sections were probed using HA (1:800, Cell Signaling Technologies, #3724) and anti-rabbit antibodies (1:1000, Alexa Fluor 594, Thermo Fisher #A-11012).

### von Frey filament test

Mice were placed on a suspended wire mesh, and monofilaments of increasing diameter with forces ranging from 0.008 to 11.0 g (NC Medical, catalog #NC12775-01) were pressed against the hind paw skin. Responses (withdrawal/no withdrawal) were recorded until the foot was withdrawn for 5 consecutive trials.

### Open-field testing

Mice were placed in a 24-inch x 24-inch open plexiglass box and allowed to explore freely for 10 mins. Perimeter was defined as 6 inches from any wall, and corners were defined by a 6-inch x 6-inch square. Center was defined as a 12-inch x 12-inch square in the arena center. Sessions were analyzed using Noldus Ethovision XT tracking software.

### Fear-conditioning

Day 1: Mice were habituated to context A for 10 min followed by context B for 10 min. Day 2: mice were placed in context A for 5 min and a 30 sec baseline was collected with a 65 dB tone. An 80 dB tone (conditioned stimulus, CS) was presented for 30 sec co-terminating with a 1 sec 0.6 mA shock. 3 tone-shock pairings were presented. Days 3–7: Mice were placed in context B and a 30-sec baseline was collected. The CS tone was presented for 30 sec and the baseline – CS tone presentation was repeated for 15 trials with 30-sec intertrial intervals. Movement was measured using a piezoelectric accelerometer and recorded using SR-Lab software.

### Statistical analysis

All data are presented ± SEM. Statistical measurements were performed in GraphPad Prism and RStudio, and figures were prepared in GraphPad Prism and BioRender. Details for all statistical tests are presented in figure legends and Tables S2 and S3. Outliers were determined using Grubbs’ test with alpha set to 0.05. All testing was conducted by experimenters blinded to treatment and genotype groups.

## Results

### The DREADD ligand clozapine-N-oxide effectively activates the HPA axis in CRF-Cre + /DREADD+ mice

**Fig. 1:**
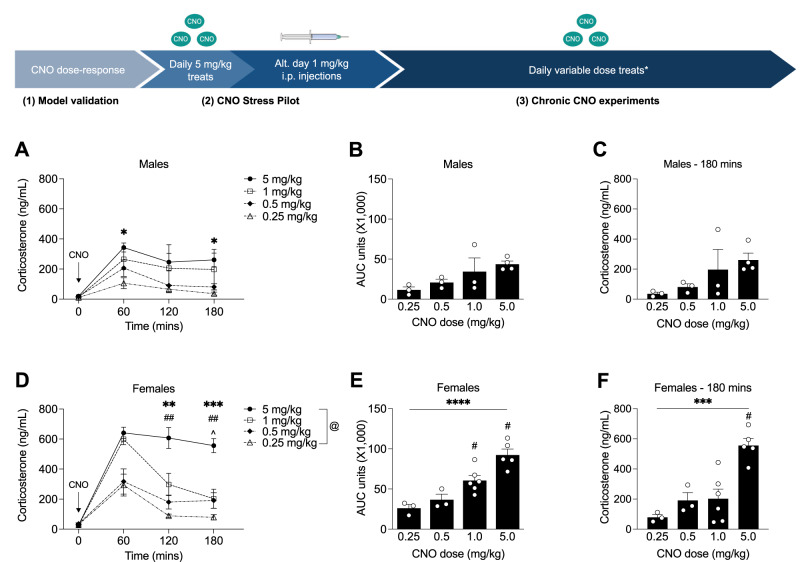
CNO induces dose-responsive corticosterone release in CRF-Cre + /DREADD+ mice. **A** Corticosterone levels were measured in response to 4 CNO doses in DREADD+ males (2-way RM ANOVA; F_time_(1.698,15.28) = 15.91, *p * < 0.001; F_dose_(3,9) = 2.933, *p * = 0.092; F_time*dose_(9,27) = 1.278, *p * = 0.293; *n* = 3–4). Corticosterone was elevated in response to 5 mg/kg CNO compared to 0.25 mg/kg CNO at 60 mins (*p * = 0.020) and 180 mins (*p * = 0.041) post-injection. **B** Area under the curve analysis of total corticosterone release did not show a significant effect of CNO dose (1-way ANOVA; F_dose_(3,9) = 2.998, *p * = 0.088; *n* = 3–4). **C** CNO dose did not significantly affect corticosterone levels at the HPA axis recovery timepoint in DREADD+ males (1-way ANOVA; F_dose_(3,9) = 2.403; *p * = 0.135; *n* = 3–4). **D** Corticosterone responses in DREADD+ females were significantly affected by CNO dose (2-way RM ANOVA; F_dose_(3,13) = 18.28, *p * < 0.0001; F_time_(2.066,26.86) = 55.10, *p * < 0.0001; F_time*dose_(9,39) = 6.684, *p * < 0.0001; 120 mins post-injection: 5 mg/kg vs 0.5 mg/kg *p * = 0.009; 5 mg/kg vs 0.25 mg/kg *p * = 0.005; 180 mins post-injection: 5 mg/kg vs 1 mg/kg, *p * = 0.007; 5 mg/kg vs. 0.5 mg/kg *p * = 0.013; 5 mg/kg vs 0.25 mg/kg *p * = 0.0008; *n* = 3–6). **E** CNO dosing significantly affected the total amount of corticosterone released in DREADD+ females (1-way ANOVA; F_dose_(3,13) = 17.04, *p * < 0.0001; 0.25 mg/kg vs. 1.0 mg/kg *p * = 0.021; 0.25 mg/kg vs. 5.0 mg/kg *p * = 0.0001; 0.5 mg/kg vs. 5.0 mg/kg *p * = 0.0006, 1.0 mg/kg vs. 5.0 mg/kg *p * = 0.013; *n* = 3–6). **F** 5 mg/kg CNO significantly elevated corticosterone at the HPA axis recovery timepoint in DREADD+ females (1-way ANOVA; F_dose_(3,13) = 13.51, *p * = 0.0003; 5 mg/kg vs 1.0 mg/kg *p * = 0.001; 5 mg/kg vs. 0.5 mg/kg *p * = 0.005; 5 mg/kg vs 0.25 mg/kg *p * = 0.0005; *n* = 3–6). (**** *p * < 0.0001, ***p  < 0.001, **p  < 0.01, ^##^*p * < 0.01, *p  < 0.05, ^*p * < 0.05, ^#^*p * < 0.05, @ main effect of genotype). *An example week of the variable-dose CNO treat paradigm is shown in Table S1.

To validate that chemogenetic CRF neuron activation initiates an HPA stress axis hormonal response and to determine optimal CNO dosing to produce physiologically relevant responses, we injected CNO i.p. at doses from 0.25 to 5 mg/kg in adult male and female DREADD+ mice and measured plasma corticosterone levels. Males showed increased corticosterone in response to a 5 mg/kg dose compared to a 0.25 mg/kg dose (Fig. 1A) at 60 min and 120 min following CNO injection. Area under the curve analysis (Fig. 1B) showed a trend of increasing corticosterone release with increasing CNO dose that did not rise to the level of statistical significance, likely due to the small N’s and blunted HPA axis response of C57BL/6 J mice. We did not find a significant effect of CNO dose on the HPA response recovery timepoint in males (Fig. 1C). In females, a 5 mg/kg CNO dose induced higher corticosterone release at 120- and 180-mins post-injection (Fig. 1D). Area under the curve analysis (Fig. 1E) showed a significant effect of CNO dose on the total amount of corticosterone released and a significantly prolonged HPA response at the 5 mg/kg dose (Fig. 1F). We additionally confirmed that 1 mg/kg of CNO did not induce a corticosterone response in DREADD- males (Fig. S1A) or DREADD- females (Fig. S1B).

### Repeated high-dose CNO administration induces significant stress-like physiological changes in DREADD+ male mice

**Fig. 2:**
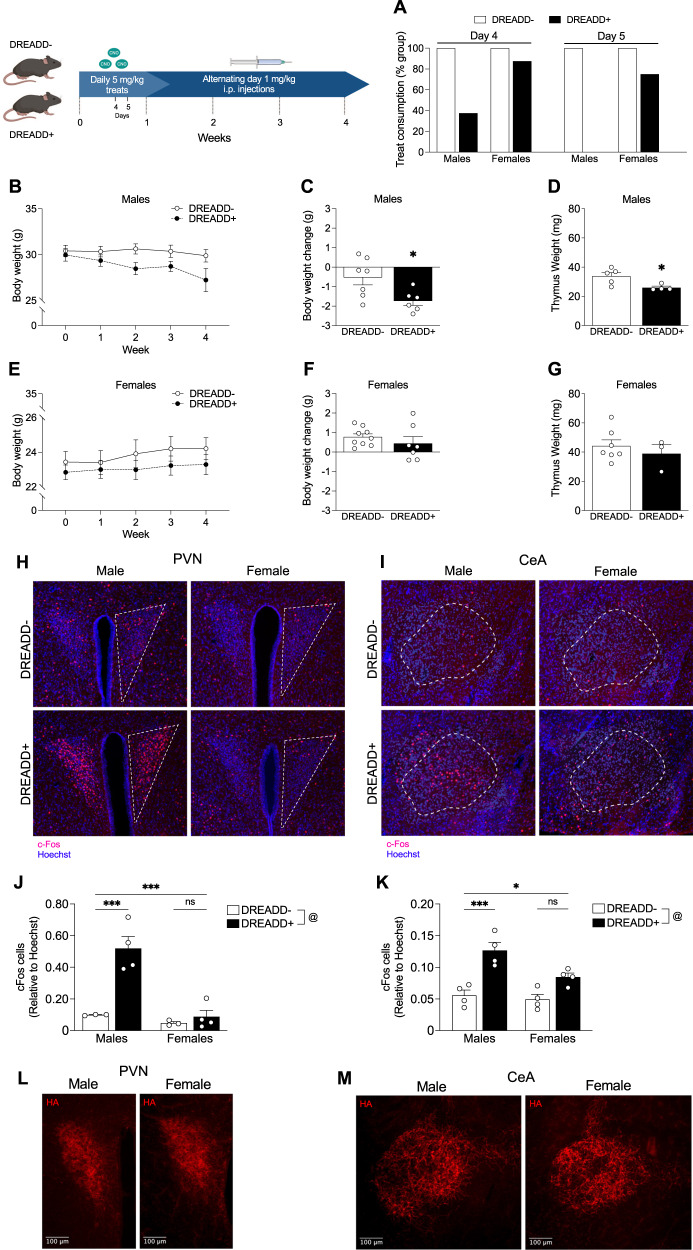
DREADD+ males display physiological stress features and have increased c-Fos expression in the PVN and central amygdala following repeated high-dose CNO. **A** 4 days after beginning CNO treat consumption, 62.5% of DREADD+ males (*n* = 7) partially or fully stopped consuming CNO treats compared to 12.5% of female DREADD+ mice (*n* = 9). 100% of DREADD+ males partially or fully stopped consuming the treats after 5 days compared to 25% of DREADD+ females. **B** Weekly body weight measurements across 4 weeks of CNO in male mice (2-way RM ANOVA; F_Time_(1.345,16.14) = 4.716, *p * = 0.039; F_genotype_(1,12) = 3.201, *p * = 0. 099; F_time*genotype_(4,48) = 2.480, *p * = 0.056; *n* = 7 per group). **C** 4 weeks of CNO significantly reduced body weight (unpaired t-test, t_(11)_ = 2.638, *p * = 0.023; *n* = 6–7) and **D** thymus weights (unpaired t-test; t_(11)_ = 2.638, *p * = 0.023, *n* = 4–5) in DREADD+ males compared to controls. **E** Weekly body weight measurements across 4 weeks of CNO in female mice (2-way RM ANOVA; F_time_(2.478,34.70) = 4.938, *p * = 0.009; F_genotype_(1,14) = 0.751, *p * = 0.401; F_time*genotype_(4,56) = 0.964, *p * = 0.435; *n* = 7–9). **F** 4 weeks of CNO did not induce overall body weight change (unpaired t-test, t_(14)_ = 0.952, *p * = 0.357, *n* = 7–9) or (**G**) affect thymus weights (unpaired t-test; t_(8)=_0.721, *p * = 0.492; *n* = 3–7) in female DREADD+ mice compared to controls. **H** Representative images of c-Fos immunostaining in the PVN and (**I**) central amygdala. Dashed lines indicate region of interest used for quantification. **J** Quantification of c-Fos immunoreactivity in the PVN (2-way ANOVA; F_sex_(1,10) = 22.77, *p * = 0.0008; F_genotype_(1,10) = 20.68, *p * = 0.001, F_sex*genotype_(1,10) = 14.15, *p * = 0.004; *n* = 3–4). DREADD+ males had a significantly higher proportion of c-Fos immunoreactivity compared to controls (*p * = 0.0007) and DREADD+ females (*p * = 0.0003) while there were no significant differences in DREADD+ females compared to controls (*p * = 0.943). **K** Quantification of c-Fos immunoreactivity in the CeA (2-way ANOVA; F_sex_(1,12) = 7.037, *p * = 0.021; F_genotype_(1,12) = 34.16, *p * < 0.0001; F_sex*genotype_(1,12) = 3.831, *p * = 0.074). DREADD+ males had a significantly higher proportion of c-Fos immunoreactivity (*p * = 0.0007) compared to controls and DREADD+ females (*p * = 0.03) while there were no significant differences in c-Fos immunoreactivity between DREADD+ females and controls (*p * = 0.073). **L** Representative images of HA immunostaining in the PVN and (**M**) CeA in male and female DREADD+ mice. By visual inspection of expression patterns, no apparent differences were noted. (***p  < 0.001, *p  < 0.05, @ main effect of genotype).

After confirming that CNO effectively induced acute stress-like corticosterone release in DREADD+ mice, we next wanted to determine if repeated CNO administration would replicate known stress-relevant physiological phenotypes. To minimize both experimenter time and handling stress to control animals, we used highly palatable cookie dough treats containing a measured dose of CNO. We administered a single 5 mg/kg CNO treat daily, and remarkably, by day 5, all male DREADD+ mice partially or fully stopped consuming CNO treats compared to 25% of female DREADD+ mice (Fig. 2A). To complete this initial study, we transitioned to 1 mg/kg CNO alternating day i.p. injections. Body weight loss and thymus atrophy are physiological hallmarks of severe stress, and we found that male DREADD+ mice showed a trend of decreasing body weight across 4 weeks (Fig. 2B) and lost significantly more weight (Fig. 2C) and had significantly smaller thymuses at the end of 4 weeks (Fig. 2D). Interestingly, female DREADD+ mice did not show body weight changes (Fig. 2E) or have significantly different body weights (Fig. 2F) or thymus weights (Fig. 2G) at the end of 4 weeks.

To determine whether these sex-specific effects were due to sex differences in CRF neuron activation, we measured c-Fos immunoreactivity in both the PVN and central amygdala (CeA) in response to CNO, two regions with high CRF neuron densities (Fig. 2H, I). In the PVN, DREADD+ males had a significantly higher proportion of c-Fos immunoreactivity while DREADD+ females were not significantly different from controls (Fig. 2J). In the CeA, DREADD+ males again had a significantly higher proportion of c-Fos immunoreactivity while there were no significant differences in c-Fos immunoreactivity between DREADD+ females and controls (Fig. 2K). To confirm that the DREADD receptor is expressed similarly in CRF neurons between males and females, we performed immunohistochemistry using the HA-tag on hM3Dq. Based on visual inspection of expression patterns, no apparent differences were observed in either the PVN (Fig. 2L) or CeA (Fig. 2M).

### 9 weeks of variable lower-dose CNO does not induce a severe stress phenotype in DREADD+ mice

**Fig. 3:**
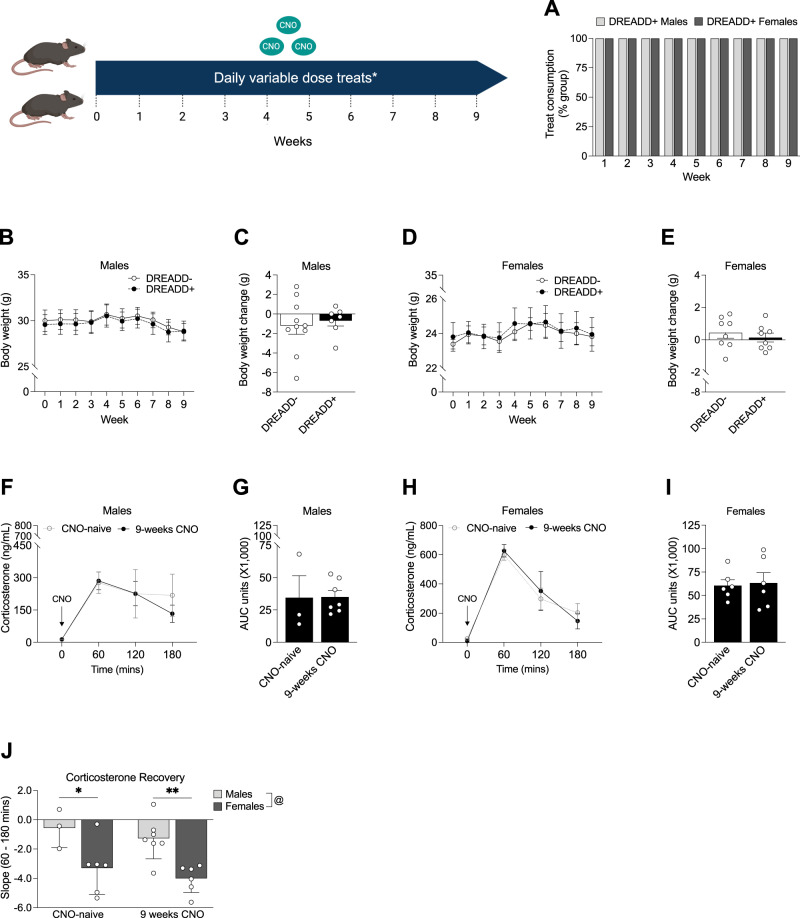
Lower variable-dose chronic CNO does not induce a severe stress physiological phenotype or lead to CNO habituation. *An example week of the variable-dose CNO treat paradigm is shown in Table S1. **A** 100% of male (*n* = 7) and female (*n* = 8) DREADD+ mice continued consuming daily CNO treats across 9 weeks. **B** Weekly body weight measurements across 9 weeks in DREADD- (*n* = 10) and DREADD+ (*n* = 7) males (2-way RM ANOVA; F_time_(1.091,16.36) = 5.050, *p * = 0.036, F_genotype_(1,15) = 0.043, *p * = .838; F_time*genotype_(9,135) = 0.162, *p * = 0.997). **C** Lower, variable dose CNO treats did not induce a greater overall body weight change in DREADD+ males compared to controls (unpaired t-test; t_(15)=_0.434, *p * = 0.670). **D** Weekly body weight measurements across 9 weeks in DREADD- (*n* = 8) and DREADD+ (*n* = 8) females (2-way RM ANOVA; F_time_(1.230,17.22) = 2.238, *p * = 0.15; F_genotype_(1,14) = 0.039, *p * = 0.847; F_time*genotype_(9,126) = 0.151, *p * = 0.998). **E** There were no differences in overall body weight change at the end of 9 weeks in DREADD+ females compared to controls (unpaired t-test; t_(14)=_0.684, *p * = 0.505; *n* = 8 per group). **F** 9 weeks of CNO did not affect HPA axis reactivity to an acute CNO injection (2-way RM ANOVA; F_CNO chronicity_(1,9) = 0.112, *p * = 0.746; F_time_ (1.564, 14.08) = 13.09, *p * = 0.001; F_time*CNO chronicity_(3, 27) = 0.494, *p * = 0.690) or (**G**) total amount of corticosterone released following an acute CNO injection in DREADD+ males (*n* = 7) compared to CNO-naïve males (*n* = 3) (unpaired t-test; t_(8)_ = 0.0467, *p * = 0.964). **H** 9 weeks of chronic CNO did not affect HPA axis reactivity to an acute CNO injection (2-way RM ANOVA; F_CNO chronicity_(1,10) = 0.002, *p * = 0.967; F_time_(1.686, 16.86) = 40.77, *p * < 0.0001; F_time*CNO chronicity_(3, 30) = 0.379, *p * = 0.769) or (**I**) total amount of corticosterone released in female DREADD+ mice (*n* = 6) compared to CNO-naïve females (*n* = 6) (unpaired t-test; t_(10)_ = 0.221, *p * = 0.829). **J** Slope analysis of HPA axis corticosterone response from 60- to 120- mins post-injection. Female DREADD+ mice recovered faster from peak corticosterone levels than DREADD+ males when CNO-naïve (*p * = 0.014) and after 9 weeks of chronic CNO administration (*p * = 0.003) (2-way ANOVA; F_sex_(1,18) = 18.49, *p * = 0.0004; F_CNO chronicity_(1,18) = 1.231, *p * = 0.282; F_sex*CNO chronicity_(1,18) < 0.0001, *p * = 0.999). (*p  < 0.05, @ main effect of sex).

Our pilot study produced a more severe stress phenotype than we sought to model, so to avoid inducing this phenotype and to prevent DREADD+ animals from developing treat avoidance, we instead used a randomized lower-dose CNO treat paradigm consisting of 0.25 mg/kg, 0.5 mg/kg, and 1.0 mg/kg and 3 separate dough flavors to examine the effects of chronic CRF neuron activation (Table S1). Under this modified regimen, all DREADD+ mice of both sexes continued consuming the treats across 9 weeks of daily administration (Fig. 3A). In DREADD+ males, we found no significant changes in body weight across 9 weeks (Fig. 3B) and no overall body weight change after 9 weeks (Fig. 3C). Likewise, chronic CNO did not affect body weight in female DREADD+ mice (Fig. 3D) or induce overall body weight change after 9 weeks (Fig. 3E).

We next measured plasma corticosterone levels following a single 1 mg/kg CNO injection after 9 weeks of chronic CNO to determine whether repeated activation of hM3Dq in CRF neurons induced CNO habituation. Male DREADD+ animals showed no change in their HPA axis response to a CNO injection compared to CNO-naïve animals that received a 1 mg/kg injection in our initial validation experiment (Figs. 1A and 3F) and no differences in total corticosterone released (Figs. 1B and 3G). Female DREADD+ animals also showed no change in their HPA axis response following chronic CNO compared to CNO-naïve animals (Figs. 1D and 3H) and showed no differences in total corticosterone released (Figs. 1E and 3I). Both CNO-naïve and chronically treated female DREADD+ mice had faster HPA axis recovery rates than CNO-naïve and chronically treated DREADD+ males (Fig. 3J).

Many studies using classical chronic stress models report locomotive changes which led us to perform an open-field test. We found no differences between DREADD+ males and controls in distance traveled (Fig. S2A), center time (Fig. S2B), total movement time (Fig. S2C), or average velocity (Fig. S2D). Likewise, we found no differences between DREADD+ females and controls in distance traveled (Fig. S2E), center time (Fig. S2F), total movement time (Fig. S2G), or average velocity (Fig. S2H).

### Chronic CRF neuron activation increases stress reactivity in DREADD+ males, heightens fear responses in DREADD+ females, and increases tactile sensitivity in both sexes

**Fig. 4:**
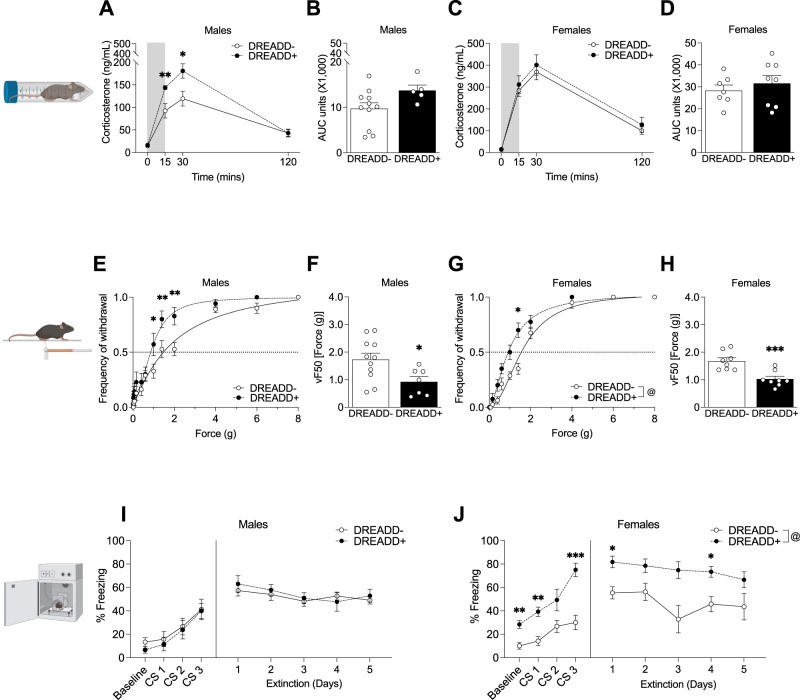
Male DREADD+ mice have heightened HPA axis reactivity while female DREADD+ mice have heightened fear responses following chronic CNO. **A** Male DREADD+ mice (*n* = 5) had an elevated HPA axis response to 15 min of restraint stress following 9 weeks of CNO (2-way RM ANOVA; F_genotype_(1,14) = 3.904, *p * = 0.068, F_time_(1.671,23.39) = 77.76, *p * < 0.0001; F_time*genotype_(3,42) = 5.130, *p * = 0.004) with a significant elevation in corticosterone levels 15 mins (*p * = 0.009) and 30 mins (*p * = 0.020) after restraint onset compared to controls (*n* = 11). **B** Area under the curve analysis did not show a significant difference in total corticosterone released in DREADD+ males following 9 weeks of chronic CNO (unpaired t-test; t_(14)_ = 1.921, *p * = 0.075). **C** 9 weeks of chronic CNO did not alter the HPA axis response to acute restraint stress in DREADD+ females (*n* = 8) compared to controls (*n* = 7) (2-way RM ANOVA; F_genotype_(1,13) = 0.529, *p * = 0.480; F_time_(2.096,27.27) = 78.92, *p * < 0.0001; F_time*genotype_(3,39) = 0.167, *p * = 0.918) and (**D**) did not affect total corticosterone released (unpaired t-test; t_(g13)_=0.729, *p * = 0.479). **E** Using the von Frey filament test to measure tactile sensitivity, DREADD+ males (*n* = 7) showed a leftward shift of the paw withdrawal curve compared to controls (*n* = 11) (2-way RM ANOVA; F_genotype_(1,16) = 4.034, *p * = 0.062; F_force_(14,224) = 169.8, *p * < 0.0001; F_genotype*force_(14, 224) = 2.658, *p * = 0.001) with DREADD+ males having significantly more paw withdrawals at 1.0 g (*p * = 0.021), 1.4 g (*p * = 0.006), and 2.0 g (*p * = 0.001) of force. **F** DREADD+ males had a lower average force required for 50% paw withdrawal (VF50) compared to controls (unpaired t-test; t_(16_) = 2.489, *p * = 0.024). **G** DREADD+ females (*n* = 8) also showed a leftward shift of the paw withdrawal curve compared to controls (*n* = 8) (2-way RM ANOVA; F_genotype_(1,14) = 14.93, *p * = 0.002; F_force_(4.396,61.55) = 371.2, *p * < 0.0001; F_genotype*force_(14,196) = 5.182, *p * < 0.0001) with significantly more paw withdrawals at 1.4 g of force (*p * = 0.014), and (**H**) had lower VF50 (unpaired t-test; t_(14)_ = 4.235, *p * = 0.0008) compared to controls. **I** Freezing behavior during auditory fear conditioning. Male DREADD+ mice (*n* = 7) showed no differences in freezing behavior during conditioning (2-way RM ANOVA; F_genotype_(1,16) = 0.287, *p * = 0.600; F_trial_(2.114,33.82) = 12.26, *p * < 0.0001; F_genotype*trial_(3,48) = 0.085, *p * = 0.968) or extinction (2-way RM ANOVA; F_genotype_(1,16) = 0.129, *p * = 0.725; F_trial_(2.441,39.05) = 5.210, *p * = 0.007; F_trial*genotype_(4,64) = 1.073, *p * = 0.377) compared to controls (*n* = 11) following 9 weeks of CNO. **J** Chronic CNO significantly elevated freezing behavior in female DREADD+ mice during conditioning (*n* = 8 per group; 2-way RM ANOVA; F_genotype_(1,14) = 23.36, *p * = 0.0003, F_trial_(2.170, 30.38) = 25.24, *p * < 0.0001; F_genotype*trial_(3,42) = 4.20, *p * = 0.011), with DREADD+ females freezing more at baseline (*p * = 0.003) and conditioning trials 1 (*p * = 0.002) and 3 (*p * = 0.0004). Chronic CNO also increased female DREADD+ freezing behavior (*n* = 6 per group) during the extinction trials (2-way RM ANOVA; F_genotype_(1,10) = 16.23, *p * = 0.002; F_trial_(2.092, 20.92) = 2.324, *p * = 0.121; F_genotype*trial_(4,40) = 0.780, *p * = 0.545), with significantly higher levels of freezing compared to controls during extinction trials 1 (*p * = 0.022) and 4 (*p * = 0.034). (***p  < 0.001, **p  < 0.01, *p  < 0.05, @ main effect of genotype).

To determine whether chronic CRF activation altered HPA axis reactivity to an acute stressor, we measured plasma corticosterone levels in response to restraint stress. Male DREADD+ mice had an elevated HPA axis response to 15 min of restraint stress (Fig. 4A) following 9 weeks of CNO with a significant elevation in corticosterone levels 15 min and 30 min after restraint onset; however, we did not find a significant effect of chronic CNO on total corticosterone release (Fig. 4B). 9 weeks of CNO did not alter the HPA response to acute restraint stress in DREADD+ females (Fig. 4C) or affect total corticosterone released (Fig. 4D).

Altered sensory processing is associated with chronic stress states so we next used the Von Frey filament test to measure changes in tactile sensitivity. Male DREADD+ mice showed increased sensitivity compared to controls (Fig. 4E) and had lower force required for 50% paw withdrawal (VF50) (Fig. 4F). Likewise, DREADD+ females displayed increased sensitivity (Fig. 4G) and decreased VF50 compared to controls (Fig. 4H). These results replicate our findings that 2 weeks of chronic multimodal stress shifted the frequency of withdrawal curve leftward in both male and female mice (Fig. S3A, C) and decreased the VF50 in males and females (Fig. S3B, D) with females showing a greater percent decrease in the VF50 from baseline than males (Fig. S3E).

Chronic stress influences the acquisition and extinction of fear memories and given the key role of CeA CRF neurons in these processes, we performed auditory fear conditioning to determine whether chronic CNO affected fear memory acquisition and extinction in DREADD+ animals. Male DREADD+ mice showed no differences in freezing behavior during conditioning or extinction (Fig. 4I) following 9 weeks of CNO. In contrast, we found a significant effect of chronic CNO on freezing with DREADD+ females freezing more at baseline and conditioning trials 1 and 3, and extinction trials 1 and 4 (Fig. 4J).

## Discussion

While utilization of animal models to study the lasting effects of chronic stress has provided important insight into mechanisms underlying disease risk, variability in outcomes between and within labs, as well as the high labor effort required for such studies, can often be an obstacle for the utilization and interpretation of these models [37, 39–45, 56–61]. We developed a model using transgenic mice expressing the Gq-coupled Designer Receptors Exclusively Activated by Designer Receptors (DREADD) hM3Dq in corticotropin-releasing factor (CRF) neurons, allowing us to activate the CRF system with the DREADD ligand clozapine-N-oxide (CNO) in a timing-selective and high-throughput manner [51, 52]. The goal of this approach was to administer ‘stress’ in a precisely controlled manner while reducing the inter-animal variability often found in chronic stress paradigms. Therefore, our studies used a chemogenetic approach designed to validate expected outcomes aligned findings from decades of stress research [12, 23, 25, 32, 37, 56–74].

We first utilized the HPA stress axis to validate CNO activation of hM3Dq-expressing CRF neurons. As predicted, CNO activated the HPA axis in DREADD+ mice, increasing corticosterone levels with a time course similar to an acute stressor [53, 75, 76]. The CNO activation of CRF neurons appeared dose-dependent. Interestingly, dose-dependent glucocorticoid production following modulation of CRF neuron activity has not been previously shown [77, 78]. Our results demonstrate that directly modulating CRF neuron activity is sufficient to alter the degree of HPA axis activation and ultimately direct corticosterone levels, highlighting a unique aspect of this model whereby the severity of ‘stress’ can be controlled by CNO dose. We also reproduced sex differences in peak corticosterone production that mirrored known sex differences in adult rodent HPA reactivity [79–84]. Interestingly, females had faster recovery rates than males at all doses tested except the highest.

Our ultimate goal was to model chronic stress with this chemogenetic approach. CNO is typically administered via intraperitoneal (i.p.) injection; however, this method produces animal stress and pain, including to control animals, and is not high throughput. Therefore, we developed CNO-containing cookie dough treats that could be administered efficiently, consumed within a defined timeframe, and be easily replicated across labs. As the bioavailability and metabolism of oral CNO was not clear, we began this pilot study with a daily dose of 5 mg/kg [85–87]. Interestingly, within the first 5 days of daily CNO treat consumption, all DREADD+ male mice, but only 25% of DREADD+ female mice, partially or fully stopped consuming the treats. To determine if this behavior reflected an anhedonic state resulting from the effects of repeated CRF activation, we administered palatable fruit-flavored sucrose pellets to the mice and found that all mice readily consumed these treats. This suggested that males learned the negative association faster or were more sensitive to the negative effects of CRF activation. Interestingly, this mirrors findings from conditioned taste aversion studies where male rodents developed an aversion to lithium chloride-containing saccharin faster than females and were slower to extinguish this aversion [88–92].

To continue this pilot study, we transitioned from oral CNO treats to alternate-day i.p. injections. After 4 weeks, males, but not females, lost body weight, a classic physiological sign of severe stress [70, 93–95]. Similarly, males, but not females, showed the classic stress phenotype of decreased thymus weight, consistent with known stress-induced thymus atrophy produced by elevated glucocorticoids [58, 96–98]. It was notable that despite having a lower corticosterone response to CNO than females, males developed CNO treat avoidance faster and displayed physiological features of severe stress. Glucocorticoids are primary mediators of stress effects, and many rodent studies model stress through direct corticosterone administration [99–106]. Our results, however, suggest an interesting sex-specific dissociation between glucocorticoid production and physiological outcomes, supporting possible cellular- and tissue-specific processes controlled by glucocorticoids.

Next, to examine possible sex differences in CRF neuron activation, we measured c-Fos immunoreactivity as a proxy for neuronal activity in 2 brain regions with significant CRF neuron populations, the PVN and central amygdala (CeA). 3 h following a CNO injection, male DREADD+ mice showed significantly more c-Fos immunoreactivity in both brain regions compared to DREADD+ females. Neuronal depolarization rapidly induces c-Fos expression, and persistent c-Fos immunoreactivity 3 h after CNO administration suggests either prolonged or increased neuronal activity in males in the PVN and CeA [107–109]. DREADD+ females appear to shut down CRF neuron activity more rapidly than males, though we cannot draw definitive conclusions without electrophysiological confirmation. This possibility is supported, however, by our results that females also recover faster from chemogenetic HPA axis activation at this same CNO dose. As we directly activated CRF neurons with CNO, it is not clear if these findings translate to all types of stress experiences.

Chronic stress is a critical underlying risk factor for future neuropsychiatric disease [31, 67, 70–72, 110, 111]. Our goal for developing this model was to titrate CRF activation to mimic chronic environmental stress without producing severe stress, and our 4-week pilot CNO data suggested that daily 5 mg/kg treats and even 1 mg/kg dosing every other day produced a more severe phenotype than we wanted to model. Therefore, we modified our CNO treat paradigm for a chronic study to include multiple flavors and 3 alternating CNO doses. Over 9 weeks of daily treatment, we found no changes in body weight, and all mice continued to consume daily CNO treats, supporting that we had identified a paradigm that was effective in both sexes and did not induce severe physiological stress phenotypes. Using an acute i.p. injection following 9 weeks of treatment, we validated that both male and female DREADD+ mice continued to respond to CNO in a nearly identical manner to CNO-naïve animals, indicating that DREADD-expressing CRF neurons remained responsive to CNO. Taken together with the more severe stress phenotypes induced by higher dose CNO in our pilot study, these results again highlight an advantage of this model where CNO dosing can induce a wide range of desired stress phenotypes.

Many neuropsychiatric diseases are characterized by altered HPA stress responses, and preclinical studies have repeatedly demonstrated that chronic stress alters HPA axis reactivity to acute stressors [112–122]. Our approach allowed us to ask whether these changes were encoded at the level of the CRF neurons themselves or by changes in afferent drive. In response to acute restraint stress following 9 weeks of chronic CRF activation, DREADD+ males showed a significant elevation in their HPA axis response compared to DREADD- males, but no difference was observed in females. These data suggest that the effects of chronic stress may be encoded upstream of CRF neurons. Indeed, chronic stress decreases PVN CRF neuron inhibition through both reduced GABAergic signaling and decreases endocannabinoid-mediated negative feedback, and increases glutamatergic and noradrenergic excitation [69, 73, 123–126].

Stress-mediated changes in sensory sensitivity are a common and translatable measure [127–129]. Therefore, we used the von Frey filament test to measure tactile sensitivity. We found that both male and female DREADD+ mice showed a leftward shift of the withdrawal curve and decreased VF50 following 9 weeks of CNO, indicating elevated tactile sensitivity. These results replicate our findings that both male and female mice showed leftward withdrawal curve shifts and decreased VF50 after 2 weeks of conventional multimodal stress. Chronic stress and enhanced sensory sensitivity have a bidirectional relationship, and the heightened tactile perception highlights a vulnerability of the somatosensory system to chronic stress.

We next utilized auditory fear conditioning to determine whether chronic CRF activation altered fear memory acquisition and extinction. Limbic CRF neurons, including in the CeA, have an important role in the formation and extinction of fear memories. [13, 15, 35, 36, 64, 65, 130–139]. Following 9 weeks of CNO, female DREADD+ mice froze significantly more than controls at baseline and across all conditioning and extinction trials, while male DREADD+ mice showed no differences in freezing compared to controls. Inbred mouse strains often do not show robust fear extinction using this paradigm of extinction trials, making it difficult to evaluate between groups [140–144]. However, the female-specific heightened freezing, even prior to foot shock, suggests that chronic CRF activation uniquely sensitizes fear responses in females and may reflect underlying female-specific vulnerability. Certainly, in humans, females are more than twice as likely as males to develop PTSD following traumatic events [145–151]. Interestingly, the sex differences in fear conditioning mirrored those seen in corticosterone production, i.e., females produced higher levels of corticosterone and showed heightened freezing. While glucocorticoids feedback negatively on PVN CRF neurons, they have a positive relationship with CeA CRF neurons [23, 152, 153]. Further studies are needed to dissect the mechanisms underlying the potential sex-specific sensitivities of CRF populations, including the PVN and CeA.

There are several limitations to this work that could be examined in future studies. First, the simultaneous activation of CRF-neurons in the brain for an extended period may not completely recapitulate the time course of circuit activity under a natural stress exposure [74, 109, 152, 154, 155]. While the majority of parvocellular PVN AVP neurons co-express CRF and therefore would be activated in our model, we did not examine AVP contributions or ACTH levels in these studies [156–158]. These studies largely focused on outcomes directly attributed to the PVN and CeA, however, additional CRF neuronal populations have important roles in orchestrating the stress response, including the bed nucleus of the stria terminalis (BNST) and the basolateral amygdala (BLA) [17–20, 138, 159]. Future work should examine the roles of these additional populations in the development of chronic stress-relevant phenotypes. Lastly, for several reasons outside our control (e.g., pandemic, vivarium parasite infestation), our experimental mouse numbers for some of the early pilot experiments were lower than a power calculation would recommend for detecting statistically significant sex differences. Direct examination of sex differences will be critical for future mechanistic studies. Our findings also highlight a need to re-examine the methods used for measuring stress-induced phenotypes. The robust differences we found in freezing and increased tactile sensitivity suggest that the addition of sensory tests to behavioral measures may enhance our ability to detect more nuanced and sex-specific phenotypes. Collectively, these results demonstrate the potential advantages of this model utilizing CNO to acutely or chronically activate hm3Dq-expressing CRF neurons to examine sex-specific stress-relevant phenotypes related to neuropsychiatric disorders.

### Supplementary information

Supplemental Material
