# Supplementary material for: Chemogenetic activation of CRF neurons as a model of chronic stress produces sex-specific physiological and behavioral effects
Source: Neuropsychopharmacology. 2023 Oct 13;49(2):443–54. doi: 10.1038/s41386-023-01739-5 (PMC10724197; doi:10.1038/s41386-023-01739-5)
Supplement: Supplementary file 1 — Supplemental Material [file 41386_2023_1739_MOESM1_ESM.pdf]

**Supplementary Materials and Methods**

*Animals*

B6N;129-Tg(CAG-CHRM3*,-mCitrine)1Ute/J mice (Jackson Laboratories, strain #026220) were crossed with B6(Cg)-Crhtm1(cre)Zjh/J mice (Jackson Laboratories, strain #012704) mice to generate animals that were either heterozygous for both the CRF-Cre transgene and the hM3Dq transgene (CRF-Cre$^{+/-}$ X DREADD$^{+/-}$, defined as DREADD+) or heterozygous for the CRF-Cre transgene and wild-type for the hM3Dq transgene (CRF-Cre$^{+/-}$ X DREADD$^{-/-}$, defined as DREADD-). Mice were housed on a 12-hr light:dark cycle and food (PicoLab irradiated rodent diet, catalog #5L0D) and water were provided *ad libitum*. Adult (10-20 weeks) male and female DREADD+ and DREADD- mice were used for all DREADD studies. Adult (8-9 week) male and female C57BL/6J (Jackson Laboratories, strain #000664) mice were used for chronic multimodal stress to assess the effects of chronic stress using the von Frey filament test. Estrous cycle was not synchronized across female subjects. Animals were singly housed for the duration of experiments. Body weights were collected weekly within 4 hrs of lights on. All animal experiments were approved by the University of Maryland Baltimore Institutional Animal Care and Use Committee and conducted in accordance with the National Institutes of Health Guide for the Care and Use of Laboratory Animals.

*CNO administration*

For i.p. injections, clozapine N-oxide (CNO) dihydrochloride (Hello Bio, catalog #HB6149) was dissolved in 0.9% saline at a concentration of 1 mM. For cookie dough treats, CNO was dissolved in Milli-Q water at a concentration of 1 mM and mixed into cookie dough (Transgenic Dough Diet, bacon flavor, Bio-Serv catalog #S3472) at a concentration of 0.1 mg CNO/g dough. To ensure an even distribution of CNO in the dough, blue food coloring (Wilton) was added to dissolved CNO before mixing with dough and forming pellets. 5 mg/kg treats were provided daily for the pilot study, however, the DREADD+ mice began to avoid the CNO treats which led us to use 1 mg/kg

i.p. injections every other day for the remainder of the pilot study. For the 9-week chronic stress study, 3 separate flavors of cookie dough treats (bacon, sugar cookie (Pillsbury), and Reese's Peanut Butter (Pillsbury)) were prepared as described above. 3 doses (0.25, 0.5, or 1 mg/kg) of the cookie dough treats were randomized daily across 9 weeks, where each mouse received a single treat each day and doses were repeated every 3 days (see example week dosing schedule in Table S1.) Both DREADD+ and DREADD- mice were treated with CNO in the DREADD studies. Dough was stored at -20$^{o}$C until administration, and any uneaten treat was removed after 24 hrs. A single treat was given to each mouse once daily, and treats were placed in mouse cages within 2 hrs of lights on except on behavioral testing days where the treat was administered after testing.

*Hypothalamic-Pituitary-Adrenal response to CNO and acute restraint stress*

For corticosterone responses to CNO, CNO was administered via i.p. injection (time 0) 1-2 hours after lights-on. A single tail snip, <1 mM from distal tip, was made at time 0 and 10 uL of blood was collected from the tail at 0-, 60-, 120-, and 180-minutes post-injection. Corticosterone response to acute restraint stress was performed 1-2 hrs after lights-on. Mice were placed in a 50 mL conical tube modified with breathing holes for 15 min and then returned to the home cage for the remainder of the test. A single tail snip, < 1 mM from distal tip, was made at time 0, and 10 uL of blood was collected at 0 and 15 minutes (onset and end of restraint, respectively) and 30- and 120-minutes following restraint onset. Blood samples from CNO administration or acute restraint stress were mixed with 5 uL of 50 mM EDTA and kept on ice and then centrifuged at 5000 rpm for 10 minutes at 4$^{o}$C. 3.02 uL plasma was collected and stored a -80$^{o}$C until corticosterone levels were measured by radioimmunoassay according to the manufacter's instructions (MP Biomedicals, catalog #07120103).

*Chronic multimodal stress*

Adult (8-9 week) male and female C57BL/6J mice were subjected to 4 hrs of stress daily from 9 am to 1 pm, for 14 days as previously described [54,55]. Stress consisted of restraint in a 3D printed restraint tube (Ender 3 printer, Creality; design available at github.com/AndreasBWulff/RestraintTube) in a cage that was tilted approximately 30$^o$ while being subjected to white noise, strobe lights, and predator odor (fox urine, Trap Shack Company, Neillsville, WI).

*Immunohistochemistry and thymus collection*

All euthanasia occurred within 4 hrs of lights on. 3 hrs after a single CNO injection, mice were anesthetized with isoflurane and transcardially perfused with ice-cold 1X PBS followed by 4% paraformaldehyde. The thymus was dissected out and weighed, and only thymuses from animals that were fully perfused were included in analyses. Brains were removed and post-fixed in 4% paraformaldehyde for 24 hours followed by 30% sucrose for 24 hours and then frozen on dry ice and stored at -80$^o$C until sectioning. 30 um slices were collected at -0.22 to -1.06 mm from Bregma for the paraventricular nucleus of the hypothalamus (PVN) and -1.22 to 2.30 mm from Bregma for the central amygdala (CeA). c-Fos immunohistochemistry was performed as described [54]. Sections were probed using a c-Fos primary antibody (1:2500, Synaptic Systems catalog #226-308, guinea pig monoclonal), anti-guinea pig secondary antibody (1:200, Alexa Fluor 568, Thermo Fisher, catalog #11075), and Hoechst counterstain (1:2000, Thermo Fisher, catalog #33342). 4X images were captured using an ECHO Revolve microscope (Bico) and Hoechst and c-Fos densities were quantified in FIJI with the Otsu method of thresholding and the Analyze Particles function as previously described [56]. Density of c-Fos was normalized to Hoechst for each section. For HA immunohistochemistry, sections were probed using HA primary antibody (1:800, Cell Signaling Technologies, catalog #3724) and anti-rabbit secondary antibody (1:1000, Alexa

Fluor 594, Thermo Fisher catalog # A-11012). 10X images were captured using an Olympus IX81 microscope. All tissue processing was done in parallel.

*von Frey filament test*

Mice were habituated to the testing room and testing apparatus for 10 min per day for 3 days prior to testing. On the testing day, following a 10-min habituation, mice were placed on a suspended wire mesh, and monofilaments of increasing diameter with forces ranging from 0.008 to 11.0 g (NC Medical, catalog #NC12775-01) were pressed against the hind paw skin. Responses (withdrawal/no withdrawal) were recorded until the foot was withdrawn for 5 consecutive trials.

*Open-field testing*

Mice were habituated to the testing room for 30 minutes prior to testing. Following habituation, mice were placed in a 24-inch x 24-inch open plexiglass box and allowed to explore freely for 10 mins within 6 hrs of lights-off. The perimeter of the testing arena was defined as 6 inches from any wall, and corners were defined by a 6-inch x 6-inch square. Center was defined as a 12-inch x 12-inch square in the center of the arena. Sessions were video-recorded and analyzed using Noldus Ethovision XT tracking software.

*Fear-conditioning*

Auditory fear conditioning was performed as described [57]. On day 1, mice were initially habituated to the chamber consisting of a non-restrictive acrylic cylinder in a sound-attenuated box (SR-LAB-Startle Response System, San Diego Instruments) in context A for 10 min followed by the chamber in context B for 10 min. Context B consisted of the original chamber modified with blue light covers and a black and white checkered pattern covering all walls and the use of 5% vinegar to clean all surfaces. Cued conditioning occurred on day 2. The animals were placed in the context A chamber for 5 min to acclimate and a 30 sec baseline was collected with a 65 dB

tone. The conditioned stimulus (CS; tone: 80 dB) was presented for 30 sec followed by the CS tone co-terminating with a 1 sec 0.6 mA foot shock (unconditioned stimulus, US). Three tone-shock pairings were presented. Extinction of the fear memory was measured on days 3-7. Mice were placed in context B and following a 5-minute acclimation period, a 30-sec baseline with the 65 dB background tone was collected. The CS tone was presented for 30 sec and the baseline – CS tone presentation was repeated for 15 trials with 30-sec intertrial intervals. Movement was measured using a piezoelectric accelerometer and recorded using the SR-Lab software.

# Supplemental Figure 1

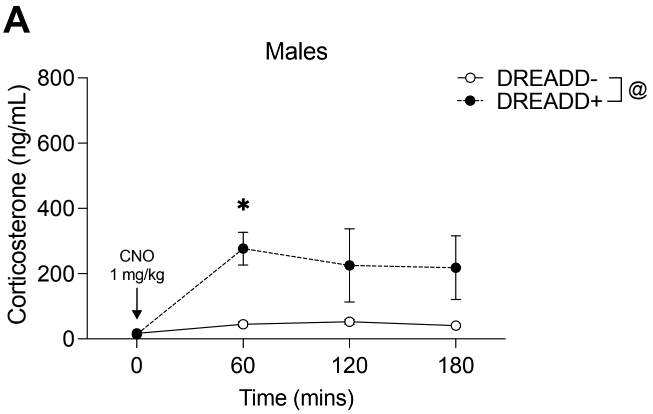

# Supplemental Figure 2

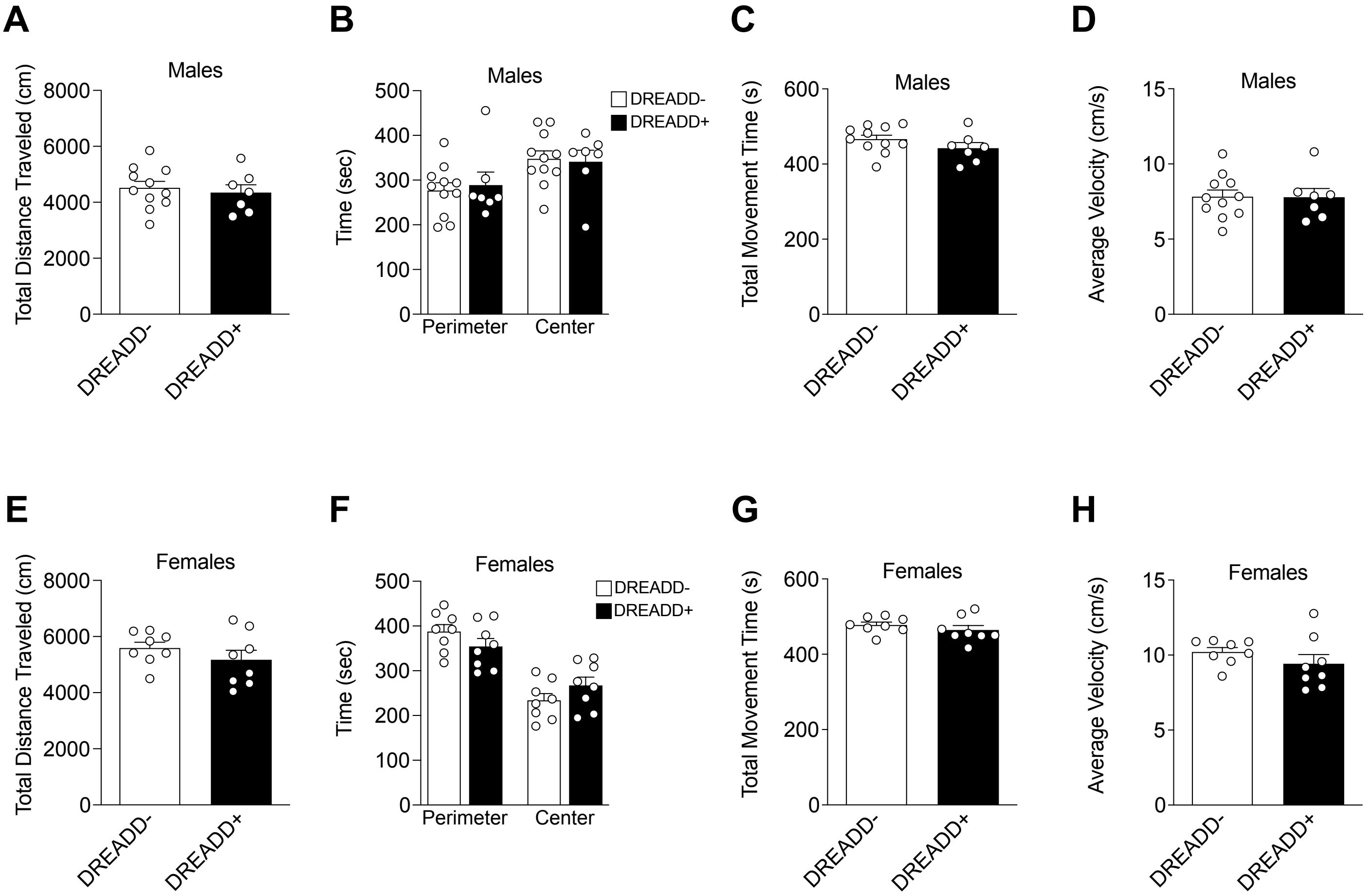

# Supplemental Figure 3

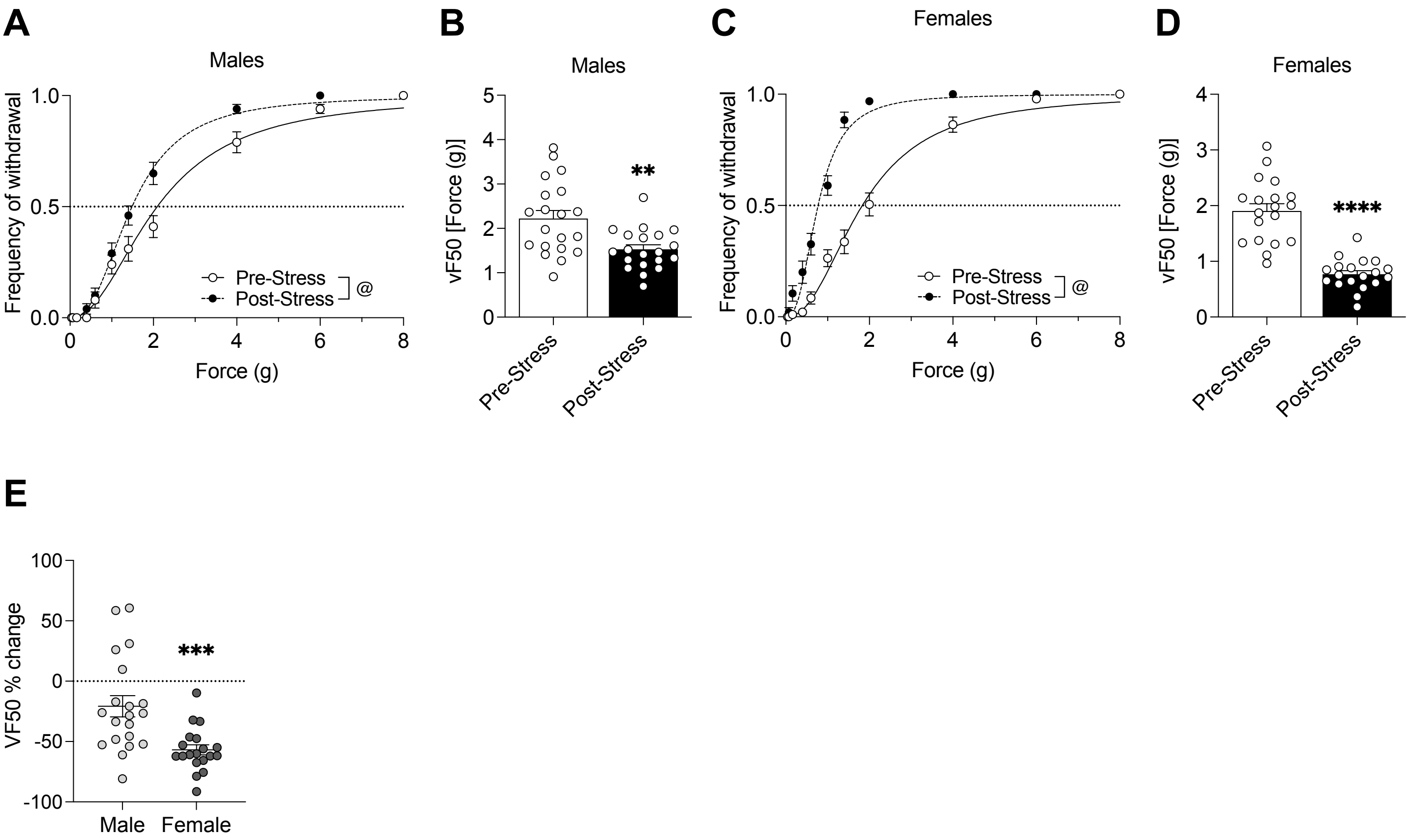

# Supplementary Table S1

| | Day 1 | Day 2 | Day 3 | Day 4 | Day 5 | Day 6 | Day 7 |
|---|---|---|---|---|---|---|---|
| **CNO Dose (mg/kg)** | 0.25 | 0.5 | 1.0 | 0.25 | 0.5 | 1.0 | 0.25 |
| **Dough flavor** | Bacon | Peanut butter | Sugar | Peanut butter | Sugar | Bacon | Sugar |

# Supplementary Table S2

| Figure | N's | Analysis | Factors | Post-hoc testing (when appropriate) | Outliers Removed (Grubb's test, alpha =0.05) | Effect Size |
|---|---|---|---|---|---|---|
| 1A | 3-4 | 2-way RM ANOVA | Time, CNO Dose | Tukey's multiple comparisons | 1 mg/kg (1) , 0.5 mg/kg (1) | $\eta^2_p$ (CNO dose) = 0.555 |
| 1B | 3-4 | 1-way ANOVA | CNO dose | N/A | None | $\eta^2$ = 0.450 |
| 1C | 3-5 | 1-way ANOVA | CNO dose | N/A | None | $\eta^2$ = 0.445 |
| 1D | 3-6 | 2-way RM ANOVA | Time, CNO Dose | Tukey's multiple comparisons | None | $\eta^2_p$ (CNO dose) = 0.750 |
| 1E | 3-6 | 1-way ANOVA | CNO dose | Tukey's multiple comparisons | None | $\eta^2$ = 0.798 |
| 1F | 3-7 | 1-way ANOVA | CNO dose | Tukey's multiple comparisons | None | $\eta^2$ = 0.757 |
| 2B | 7 per group | 2-way RM ANOVA | Time, Genotype | Tukey's multiple comparisons | None | $\eta^2_p$ (Genotype) = 0.451 |
| 2C | 6-7 | Unpaired two-tailed t test | N/A | N/A | DREADD+ (1) | Cohen's d = 1.504 |
| 2D | 4-5 | Unpaired two-tailed t test | N/A | N/A | DREADD+ (1) | Cohen's d = 1.894 |
| 2E | 7-9 | 2-way RM ANOVA | Time, Genotype | Tukey's multiple comparisons | None | $\eta^2_p$ (Genotype) = 0.447 |
| 2F | 7-9 | Unpaired two-tailed t test | N/A | N/A | None | Cohen's d = 0.459 |
| 2G | 3-7 | Unpaired two-tailed t test | N/A | N/A | None | Cohen's d = 0.500 |
| 2J | 3-4 | 2-way ANOVA | Sex, Genotype | Tukey's multiple comparisons | DREADD- male (1), DREADD- female (1) | $\eta^2_p$ (Genotype) = 0.674, $\eta^2_p$ (Sex) = 0.694 |
| 2K | 4 per group | 2-way ANOVA | Sex, Genotype | Tukey's multiple comparisons | None | $\eta^2_p$ (Genotype) = 0.740, $\eta^2_p$ (Sex) = 0.0.371 |
| 3B | 7-10 | 2-way RM ANOVA | Time, Genotype | N/A | None | $\eta^2_p$ (Genotype) = 0.030 |
| 3C | 7-10 | Unpaired two-tailed t test | N/A | N/A | None | Cohen's d = 0.226 |
| 3D | 8 per group | 2-way RM ANOVA | Time, Genotype | Tukey's multiple comparisons | None | $\eta^2_p$ (Genotype) = 0.012 |
| 3E | 8 per group | Unpaired two-tailed t test | N/A | N/A | None | Cohen's d = 0.342 |
| 3F | 4-7 | 2-way RM ANOVA | Time, CNO Chronicity | Tukey's multiple comparisons | None | $\eta^2_p$ (CNO chronicity) = 0.013 |
| 3G | 3-7 | Unpaired two-tailed t test | N/A | N/A | None | Cohen's d = 0.026 |
| 3H | 6 per group | 2-way RM ANOVA | Time, CNO Chronicity | Tukey's multiple comparisons | 10-wks CNO (2) | $\eta^2_p$ (CNO chronicity) = 0.0001 |
| 3I | 6 per group | Unpaired two-tailed t test | N/A | N/A | None | Cohen's d = 0.128 |
| 3J | 3-7 | 2-way ANOVA | Sex, CNO Chronicity | Fisher's LSD | None | $\eta^2_p$ (Sex) = 0.507, $\eta^2_p$ (CNO chronicity) = 0.064 |
| 4A | 5-11 | 2-way RM ANOVA | Time, Genotype | Fisher's LSD | DREADD+ (2) | $\eta^2_p$ (Genotype) = 0.278 |
| 4B | 5-11 | Unpaired two-tailed t test | N/A | N/A | None | Cohen's d = 1.130 |
| 4C | 7-8 | 2-way RM ANOVA | Time, Genotype | Fisher's LSD | DREADD- (1) | $\eta^2_p$ (Genotype) = 0.031 |
| 4D | 7-8 | Unpaired two-tailed t test | N/A | N/A | None | Cohen's d = 0.382 |
| 4E | 7-11 | 2-way RM ANOVA | Force, Genotype | Sidak's multiple comparisons | None | $\eta^2_p$ (Genotype) = 0.140 |
| 4F | 7-11 | Unpaired two-tailed t test | N/A | N/A | None | Cohen's d = 1.265 |
| 4G | 8 per group | 2-way RM ANOVA | Force, Genotype | Sidak's multiple comparisons | None | $\eta^2_p$ (Genotype) = 0.152 |
| 4H | 8 per group | Unpaired two-tailed t test | N/A | N/A | None | Cohen's d = 2.117 |
| 4I | 7-11 | 2-way RM ANOVA | Trial, Genotype | Sidak's multiple comparisons | None | $\eta^2_p$ (Genotype) = 0.018 (conditioning), 0.023 (extinction) |
| 4J | 6-8 | 2-way RM ANOVA | Trial, Genotype | Sidak's multiple comparisons | *Equipment malfunction led to data loss for 2 DREADD- and 2 DREADD+ subjects during extinction trial 4 | $\eta^2_p$ (Genotype) = 0.686 (conditioning), 0.553 (extinction) |
| Supp. 1A | 4-7 | 2-way RM ANOVA | Time, Genotype | Fisher's LSD | None | $\eta^2_p$ (Genotype) = 0.666 |
| Supp. 1B | 5-6 | 2-way RM ANOVA | Time, Genotype | Fisher's LSD | DREADD- (1) | $\eta^2_p$ (Genotype) = 0.749 |
| Supp. 2A | 7-11 | Unpaired two-tailed t test | N/A | N/A | None | Cohen's d = 0.228 |
| Supp. 2B | 6-11 | Unpaired two-tailed t test | N/A | N/A | DREADD + male (1) | Cohen's d = 0.104 (center), 0.364 (perimeter) |
| Supp. 2C | 7-11 | Unpaired two-tailed t test | N/A | N/A | None | Cohen's d = 0.638 |
| Supp. 2D | 7-11 | Unpaired two-tailed t test | N/A | N/A | None | Cohen's d = 0.027 |
| Supp. 2E | 8 per group | Unpaired two-tailed t test | N/A | N/A | None | Cohen's d = 0.532 |
| Supp. 2F | 8 per group | Unpaired two-tailed t test | N/A | N/A | None | Cohen's d = 0.035 (center), 0.706 (perimeter) |
| Supp. 2G | 8 per group | Unpaired two-tailed t test | N/A | N/A | None | Cohen's d = 0.473 |
| Supp. 2H | 8 per group | Unpaired two-tailed t test | N/A | N/A | None | Cohen's d = 0.575 |
| Supp. 3A | 20 | 2-way RM ANOVA | Stress, Force | Sidak's multiple comparisons | None | $\eta^2_p$ (Stress Condition) = 0.072 |
| Supp. 3B | 20 | Paired two-tailed t test | N/A | N/A | None | Cohen's d = 1.043 |
| Supp. 3C | 19 | 2-way RM ANOVA | Stress, Force | Sidak's multiple comparisons | None | $\eta^2_p$ (Stress Condition) = 0.426 |
| Supp. 3D | 19 | Paired two-tailed t test | N/A | N/A | None | Cohen's d = 2.541 |
| Supp. 3E | 19-20 | Unpaired two-tailed t test | N/A | N/A | None | Cohen's d = 1.185 |

# Supplementary Table S3

| Figure | Test | Factors | Main Effect of Sex? | $F_{(sex)}$ | P value | Interaction? | $F_{(Interaction)}$ | P value |
|---|---|---|---|---|---|---|---|---|
| 1A,D | 3-way RM ANOVA | Sex x CNO dose x Time | No | F(1, 29) = 0.3861 | P=0.3908 | (Sex x CNO Dose) Yes | F(3, 29) = 7.005 | P=0.0130 |
| 1B, E | 2-way ANOVA | Sex x CNO dose AUC | Yes | F (1, 22) = 22.74 | P<0.0001 | No | F (3, 22) = 2.261 | P=0.1096 |
| 1C, F | 2-way ANOVA | Sex x CNO dose | Yes | F (1, 22) = 6.305 | P=0.0199 | No | F (3, 22) = 2.394 | P=0.0957 |
| 2 B,E | 3-way RM ANOVA | Sex x Genotype x Time | Yes | F (1, 26) = 94.70 | P<0.0001 | (Sex x Genotype) No | F (1, 26) = 0.4210 | P=0.5222 |
| 2C, F | 2-way ANOVA | Sex x Genotype | Yes | F (1, 25) = 38.59 | P<0.0001 | No | F (1, 25) = 2.411 | P=0.1331 |
| 2D, G | 2-way ANOVA | Sex x Genotype | Yes | F (1, 15) = 8.296 | P=0.0114 | No | F (1, 15) = 0.09113 | P=0.7669 |
| 3A, C | 3-way RM ANOVA | Sex x Genotype x Time | Yes | F (1, 29) = 42.33 | P<0.0001 | (Sex x Genotype) No | F (1, 29) = 0.07686 | P=0.7836 |
| 3B, D | 2-way ANOVA | Sex x Genotype | No | F (1, 29) = 3.887 | P=0.0583 | No | F (1, 29) = 0.4061 | P=0.5290 |
| 3E, G | 3-way RM ANOVA | Sex x CNO chronicity x Time | Yes | F (1, 19) = 6.846 | P=0.0170 | (Sex x CNO Chronicity) No | F (1, 19) = 0.06600 | P=0.8000 |
| 3F, H | 2-way ANOVA | Sex x CNO chronicity | Yes | F (1, 18) = 8.911 | P=0.0079 | No | F (1, 18) = 0.01440 | P=0.9058 |
| 3I | 2-way ANOVA | Sex x CNO chronicity | Yes | F (1, 18) = 18.49 | P=0.0004 | No | F (1, 18) = 1.465e-007 | P=0.9997 |
| 4A,C | 3-way RM ANOVA | Sex x Genotype x Time | Yes | F (1, 27) = 55.47 | P<0.0001 | (Sex x Genotype) No | F (1, 27) = 0.05321 | P=0.8193 |
| 4B,C | 2-way ANOVA | Sex x Genotype | Yes | F (1, 27) = 53.61 | P<0.0001 | No | F (1, 27) = 0.02079 | P=0.8864 |
| 4E, G | 3-way RM ANOVA | Sex x Genotype x Force | No | F (1, 30) = 1.466 | P=0.2355 | (Sex x Genotype) No | F (1, 30) = 0.2428 | P=0.6258 |
| 4F,H | 2-way ANOVA | Sex x Genotype | No | F (1, 30) = 0.020 | P=0.8878 | No | F (1, 30) = 0.1611 | P=0.6910 |
| 4I,J | 3-way RM ANOVA | Sex x Genotype x Trial | Yes | F (1, 30) = 6.615 | P=0.0153 | (Sex x Genotype) Yes | F (1, 30) = 6.936 | P=0.0132 |
| 4I,J | 3-way RM ANOVA | Sex x Genotype x Trial | No | F (1, 26) = 2.560 | P=0.1217 | (Sex x Genotype) Yes | F (1, 26) = 7.677 | P=0.0102 |
| Supp. 1A,B | 3-way RM ANOVA | Sex x Genotype x Time | No | F (1, 18) = 2.974 | P=0.1017 | (Sex x Genotype) No | F (1, 18) = 2.796 | P=0.1118 |
| Supp. 2A,E | 2-way ANOVA | Sex x Genotype | Yes | F (1, 30) = 12.60 | P=0.0013 | No | F (1, 30) = 0.2247 | P=0.6389 |
| Supp. 2B,F | 2-way ANOVA | Sex x Genotype | Yes | F (1, 29) = 35.41 | P<0.0001 | No | F (1, 29) = 0.2578 | P=0.6155 |
| Supp. 2B,F | 2-way ANOVA | Sex x Genotype | Yes | F (1, 30) = 11.35 | P=0.0021 | No | F (1, 30) = 0.04628 | P=0.8311 |
| Supp. 2C,G | 2-way ANOVA | Sex x Genotype | No | F (1, 30) = 2.103 | P=0.1574 | No | F (1, 30) = 0.2067 | P=0.6527 |
| Supp. 2D,H | 2-way ANOVA | Sex x Genotype | Yes | F (1, 30) = 16.20 | P=0.0004 | No | F (1, 30) = 0.5637 | P=0.4586 |
| Supp. 3A,C | 3-way RM ANOVA | Sex x Genotype x Force | Yes | F (1, 74) = 33.54 | P<0.0001 | (Sex x Stress condition) Yes | F (1, 74) = 16.09 | P=0.0001 |
| Supp. 3B,D | 2-way ANOVA | Sex x Stress condition | Yes | F (1, 74) = 17.35 | P<0.0001 | No | F (1, 74) = 2.937 | P=0.0907 |

**Figure S1. Male and female DREADD- mice have lower corticosterone responses to CNO than DREADD+ mice. (A)** DREADD- male mice (n=7) had significantly lower corticosterone responses to 1 mg/kg of CNO than DREADD+ males (n=4) (2-way RM ANOVA, $F_{genotype}(1,9)=9.917$, $p=0.012$; $F_{time}(1.199,10.79)=10.95$, $p=0.006$; $F_{genotype*time}(3,27)=6.730$, $p=0.002$) with significantly lower levels of corticosterone at 60 mins post-injection ($p=0.018$). **(B)** DREADD- (n=5) female mice had significantly lower overall corticosterone responses to 1 mg/kg CNO than DREADD+ females (n=6) (2-way RM ANOVA; $F_{genotype}(1,9)=47.37$, $p<0.0001$; $F_{time}(1.775,15.98)=19.70$, $p<0.0001$; $F_{genotype*time}(3,27)=20.43$, $p<0.0001$) with significantly lower corticosterone at 60 mins ($p<0.0001$) and120 mins ($p=0.018$) post-injection. (****$p<0.0001$, *$p<0.05$, @ main effect of genotype).

**Figure S2. Chronic CNO does not affect locomotion in male or female DREADD+ mice.** The open field test was used to assess locomotion following chronic CNO administration. There were no differences between DREADD+ males (n=7) and controls (n=11) in **(A)** total distance traveled (unpaired t-test; $t_{(16)}=0.4697$, $p=0.646$), **(B)** center time (unpaired t-test; $t_{(16)}=0.2142$, $p=0.833$), **(C)** total movement time (unpaired t-test; $t_{(16)}=1.333$, $p=0.201$), or **(D)** average velocity (unpaired t-test; $t_{(16)}=0.058$, $p=0.954$). Likewise, there were no differences between DREADD+ females (n=8) and controls (n=8) in **(E)** total distance traveled (unpaired t-test; $t_{(14)}=1.064$, $p=0.305$), **(F)** center time (unpaired t-test; $t_{(14)}=0.070$, $p=0.945$), **(G)** total movement time (unpaired t-test; $t_{(14)}=0.950$, $p=0.358$), or **(H)** average velocity (unpaired t-test; $t_{(14)}=1.151$, $p=0.269$).

**Figure S3. Chronic multimodal stress increases tactile sensitivity in male and female mice.** Following 2 weeks of chronic multimodal stress, the Von Frey test was used to measure tactile sensitivity. **(A)** Male mice (n=20) had a significant leftward shift of the paw withdrawal curve (2-way RM ANOVA, $F_{stress}(1,38)=10.38$, $p=0.003$; $F_{force}(4.571,173.7)=422.5$, $p<0.0001$; $F_{stress*force}(10,380)=4.153$, $p<0.0001$) with significantly more paw withdrawals at 1.4g ($p=0.040$), 2.0g ($p=0.002$), 4.0g ($p=0.007$), and 6.0g ($p=0.010$) following chronic multimodal stress. **(B)** The VF50 was significantly decreased in males following chronic multimodal stress (paired t-test; $t_{(19)}=3.354$, $p=0.003$). **(C)** Female (n=19) mice also had a significant leftward paw withdrawal curve shift (2-way RM ANOVA; $F_{stress}(1,36)=68.28$, $p<0.0001$; $F_{force}(5.103,183.7)=471.2$, $p<0.0001$; $F_{stress*force}(10,360)=25.92$, $p<0.0001$) with significantly more paw withdrawals at 0.4g ($p=0.030$), 0.6g ($p=0.002$), 1.0g ($p<0.0001$), 1.4g ($p<0.0001$), 2.0g ($p<0.0001$), and 4.0g ($p=0.010$) following chronic multimodal stress. **(D)** Females also showed significantly decreased VF50 following chronic multimodal stress (paired t-test; $t_{(18)}=8.427$, $p<0.0001$). **(E)** Female mice (n=19) had a

greater percent decrease in their VF50 following chronic multimodal stress than males (n=20) (unpaired t-test; $t_{(27.17)}=3.732$, $p=0.0009$). (****$p<0.0001$, ***$p<0.001$, **$p<0.01$).
